# Optic cup and facial patterning defects in ocular ectoderm β-catenin gain-of-function mice

**DOI:** 10.1186/1471-213X-6-14

**Published:** 2006-03-15

**Authors:** Leigh-Anne D Miller, April N Smith, M Mark Taketo, Richard A Lang

**Affiliations:** 1 Division of Developmental Biology, Department of Ophthalmology, Children's Hospital Research Foundation and The University of Cincinnati, Cincinnati, OH 45229-3039, USA; 2 Department of Pharmacology, Kyoto University School of Medicine, Kyoto, Japan

## Abstract

**Background:**

The canonical *Wnt *signaling pathway has a number of critical functions during embryonic development and, when activated aberrantly, in the genesis of cancer. Current evidence suggests that during eye development, regulation of Wnt signaling is critical for patterning the surface ectoderm that will contribute to multiple components of the eye. *Wnt *signaling loss-of-function experiments show that a region of periocular ectoderm will form ectopic lentoid bodies unless the *Wnt *pathway modifies its fate towards other structures. Consistent with this, *Wnt *signaling gain of function in the ocular region ectoderm results in a suppression of lens fate.

**Results:**

Here we demonstrate that ectoderm-specific *Wnt *signaling gain-of-function embryos exhibit additional defects besides those noted in the lens. There are profound facial defects including a foreshortened snout, malformation of the nasal region, and clefting of the epidermis along the ocular-nasal axis. Furthermore, despite the restriction of *Wnt *pathway gain-of-function to the surface ectoderm, the optic cup is inappropriately patterned and ultimately forms a highly convoluted, disorganized array of epithelium with the characteristics of retina and retinal pigmented epithelium.

**Conclusion:**

We suggest that activation of the *Wnt *pathway in surface ectoderm may disrupt the normal exchange of signals between the presumptive lens and retina that coordinate development of a functional eye.

## Background

The major components of the eye are derived from two embryonic epithelial layers, the head surface ectoderm and the neurectoderm of the optic vesicle. The surface ectoderm will develop into the lens, as well as the epithelia of the cornea, conjunctiva, lacrimal gland, and Harderian gland duct [12,21,28,40,41]. The first morphological sign of lens development is the formation of the lens placode, a thickened region of the surface ectoderm that is immediately adjacent to the distal neuroepithelium of the underlying optic vesicle. After the lens placode has formed, it invaginates to generate the lens pit, a morphogenetic movement mirrored by the invagination of the optic vesicle to form the optic cup. As it invaginates coordinately with the lens pit, the optic vesicle will form inner and outer layers that respectively form the retina and retinal pigment epithelium (RPE).

In the mouse, genetic manipulations have provided significant progress towards an understanding of the molecular cascade responsible for lens induction and morphogenesis [22,35,36]. For example, the *Pax6 Small eye *alleles [14,31] and conditional deletion of *Pax6 *has shown that it is essential for lens development [1]. Furthermore, molecular epistasis studies have shown that expression of *Pax6 *in the lens placode is under the control of both *Bmp7 *[37] and *Fgf *signaling [9] suggesting that these are lens induction signals. Although it has received less attention than the lens, it has been proposed that induction of the retina is initiated by a signal from the surface ectoderm with the *Fgf *pathway being implicated [18,30]. Expression of the transcription factor Chx10 in central presumptive retina is dependent on the presence of presumptive lens ectoderm. When the presumptive lens is removed, Chx10 expression can be rescued if recombinant Fgf1 is provided [30]. *Chx10 *appears to have a role in repressing *Mitf *expression [17]. Currently, it is not understood which Fgf receptor ligands participate in either lens or retina induction.

β-catenin plays an essential role in the canonical *Wnt *pathway and, by association with cadherins, in cell-cell adhesion (reviewed in [26]). Recent analysis has shown that β-catenin has a dual function in the development of ocular surface ectoderm [32]. In the central lens region, β*-catenin *loss-of-function results in a failure of cell adhesion of lens morphogenesis, consistent with an activity in regulating cadherin function. By contrast, in the periocular ectoderm that is *Wnt *responsive, loss of β*-catenin *function results in the formation of ectopic lentoid bodies, suggesting that normally the *Wnt *pathway suppresses lens fate [32]. Mice with a null mutation in the gene for *Wnt *pathway coreceptor *Lrp6 *show defects in development of the lens epithelium at a stage when fiber cell differentiation has commenced [33]. These data suggest that there may be multiple roles for the *Wnt *pathway at different stages of lens development [27,33].

In the current study, we have used the ectoderm-specific *Lens-cre *driver [1] to assess the consequences of β*-catenin *gain-of-function in the ocular region surface ectoderm. This results in major facial abnormalities including a failure of the lens to develop and aberrant patterning of the optic cup. Interestingly, up-regulation of *Wnt *signaling in the presumptive lens ectoderm results in its acquisition of neural fate, perhaps reflecting the neural potential of head surface ectoderm. These data also emphasize the importance of signals from presumptive lens in patterning of the optic cup.

## Results and discussion

### Conditional β-*catenin *gain-of-function in embryonic head ectoderm

To assess the impact for eye development of ectoderm-specific activation of the *Wnt *pathway, we took advantage of the *Lens-cre *transgene [1] and the conditional gain-of-function β*-catenin *allele *Catnb*^*lox*(*ex3*) ^[15]. The *Lens-cre *transgene directs expression of cre recombinase to the presumptive lens and surrounding head surface ectoderm [1] using an ocular-specific enhancer (designated *EE *for ectoderm enhancer) from the *Pax6 *gene [40]. In the β*-catenin *gain-of-function allele, exon 3 is flanked by lox sites; loss of the exon 3 region of β-catenin results in a gain-of-function since the protein cannot be targeted for degradation [15]. As a first step in assessing validity of the experimental strategy and the consequences of β*-catenin *gain-of-function for eye development, we generated control embryos (with genotype *Lens-cre *or *Catnb*^*lox*(*ex3*)^) and β*-catenin *gain of-function embryos (with genotype *Lens-cre*; *Catnb*^*lox*(*ex3*)^) in combination with the *Z/AP *allele [25]. In the presence of cre recombinase, this allele switches from β*-galactosidase *(*lacz*) to *alkaline phosphatase *(*AP*) expression and so provides both a cre activity reporter and a fate-mapping tool.

**Figure 1:**
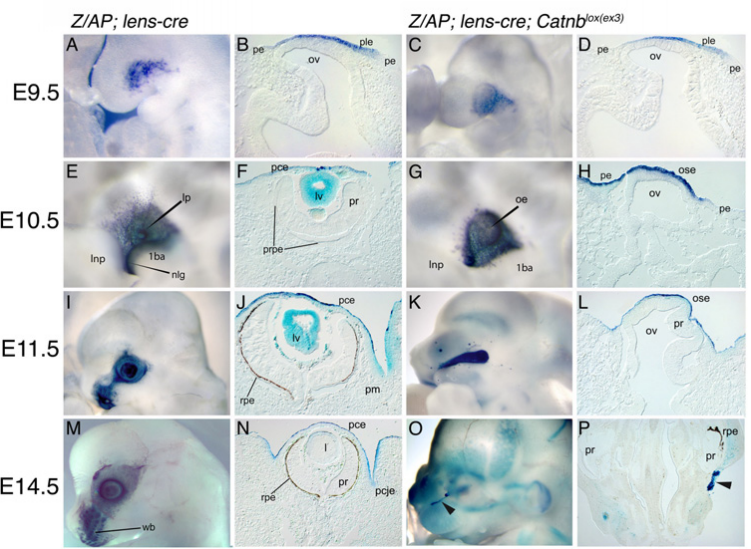
***Lens-cre *is active in ocular and nasal regions**. AP activity is shown by the blue/purple labeling in control *Lens-cre*; *Z/AP *embryos at the indicated ages either shown in whole-mount (A,E,I,M) or in section (B,F,J,N). AP activity in *Lens-cre*; *Z/AP; Catnb*^*lox*(*ex3*) ^embryos at the indicated ages either shown in whole-mount (C,G,K,O) or in section (D,H,L,P). In O and P the arrowhead points to AP staining in the naso-lacrimal groove. Labeled structures for this and all subsequent figures are as follows: ple-presumptive lens ectoderm, ov-optic vesicle, pe-periocular ectoderm, lp-lens pit, 1ba-first branchial arch, lnp-lateral nasal process, nlg-nasolacrimal groove, oe-optic eminence, pce-presumptive corneal epithelium, lv-lens vesicle, pr-presumptive retina, pm-periocular mesenchyme, ose-ocular surface ectoderm, pcje-presumptive conjuctival epithelium, rpe-retinal pigmented epithelium, prpe-presumptive retinal pigmented epithelium, wb-whisker barrels.

As anticipated [32] at embryonic day (E) 9.5, control embryos showed AP activity in the tear-drop shaped region of head surface ectoderm in which the *EE *is active (Fig. 1A). Centrally, this includes the presumptive lens ectoderm but the periocular ectoderm is also positive (Fig. 1B). In E10.5 wild-type embryos the positive region includes the lens pit, the presumptive corneal and periocular ectoderm (Fig. 1E, 1F) but has also expanded to include the ectoderm of the naso-lacrimal groove that is formed from the junction between the maxilliary component of the first branchial arch and the lateral nasal process (Fig. 1E). By E11.5, expression of *Z/AP *in the invaginating periocular surface ectoderm that will become the conjunctival epithelium has become apparent (Fig. 1I, 1J). The lens vesicle and presumptive corneal epithelium remain positive (Fig. 1J). By E14.5, the tissue specific expression pattern of AP in *Lens-cre; Z/AP *mice remains largely similar with the exception that in the nasal region, AP activity is found in the developing whisker barrels and anterior mandibular ectoderm (Fig. 1M, 1N). We conclude that the *Lens-cre *transgene has an impact on ectodermally-derived cells that are broadly distributed throughout the ocular and nasal regions.

**Figure 3:**
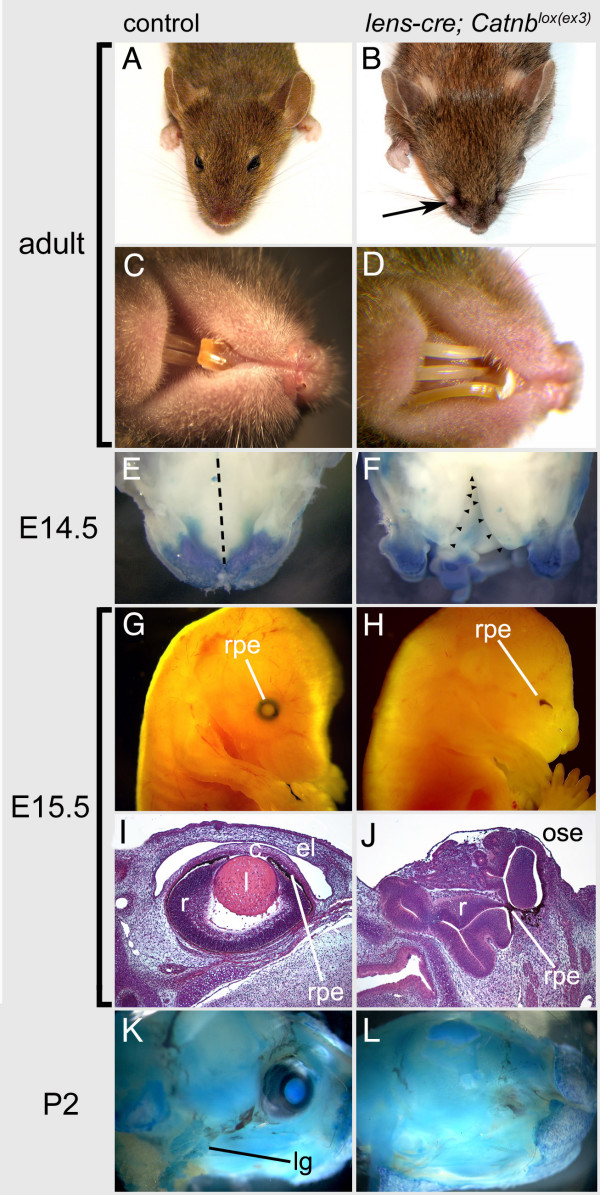
**Gross morphology of *Lens-cre*; *Catnb*^*lox*(*ex3*) ^mutant mice**. (A) Dorsal view of the head and ventral view of the mouth (C) from an adult wild-type mouse. (B) Dorsal view of the head and ventral view of the mouth (D) of an adult *Lens-cre*; *Catnb*^*lox*(*ex3*) ^mutant mouse. (E, F) Ventral view of the anterior palate in X-gal stained control (E) and *Topgal*; *Lens-cre; Catnb*^*lox*(*ex3*) ^(F) E14.5 embryos. The dashed line in (E) indicates the midline. The arrowheads in (F) show the edge of the cleft in the palate of experimental embryos. (G, H) Unstained whole mount E15.5 normal embryo (G) and *Lens-cre*; *Catnb*^*lox*(*ex3*) ^mutant embryo (H). Hematoxylin and eosin stained eye region coronal paraffin section from a control embryo (I) and *Lens-cre*; *Catnb*^*lox*(*ex3*) ^mutant embryo (J) at E15.5. (K) Nile blue staining of whole mount P2 head from control mouse and (L) from *Lens-cre;Catnb*^*lox*(*ex3*) ^mutant mouse. el-eyelids, c-cornea, l-lens, r-retina, rpe-retinal pigmented epithelium, lg-lacrimal gland, ose-ocular surface ectoderm.

This pattern of *Lens-cre *activity can largely explain the defects that arise when *Lens-cre *and *Z/AP *are combined with the *Catnb*^*lox*(*ex3*) ^gain-of-function allele. Early in eye development at E9.5, as in the wild-type, *Z/AP *expression can be detected in the presumptive lens and periocular ectoderm (Fig. 1C, 1D). By E10.5 however, there are already major defects in eye development. Under the influence of the *Catnb*^*lox*(*ex3*) ^allele, the surface ectoderm has failed to invaginate to form a lens pit and the normal morphogenesis of the first branchial arch also appears to be disrupted (Fig. 1G, 1H). By E11.5, *Z/AP *activity in *Lens-cre*; *Catnb*^*lox*(*ex3*) ^embryos is detected in a narrow band of facial ectoderm that begins caudally at the point of abnormal eye development and extends rostrally into the nasal region (Fig. 1K). At this stage, there is no indication of formation of the bilayered optic cup that is a normal feature of eye development (compare Fig. 1J with 1L). There is also no indication of invagination of the presumptive conjunctival ectoderm (Fig. 1L) and presumably as a consequence, no evidence of formation of either the lacrimal (Fig. 3J) or Harderian glands (data not shown). By E14.5, AP positive ectoderm is reduced in relative size but occupies the same ocular-nasal territory (Fig. 1O, 1P) as earlier in development. These data suggest that stabilized β-catenin has a profound affect in preventing formation of ocular and other facial structures and suggests that this occurs as a result of canonical *Wnt *pathway activation.

**Figure 2:**
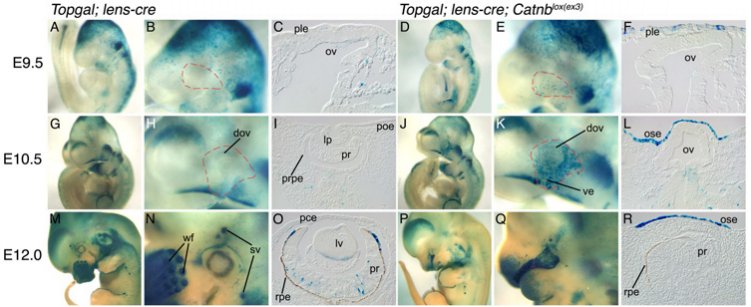
**Region of *Topgal *expression in *Lens-cre; Catnb*^*lox*(*ex3*) ^mutant embryos**. Expression of *Topgal *in control embryos at the indicated ages either shown in whole-mount (A,B,G,H,M,N) or in section for the eye region (C,I,O). Expression of *Topgal *in *Lens-cre; Catnb*^*lox*(*ex3*) ^embryos at the indicated ages either shown in whole-mount (D,E,J,K,P,Q) or in section for the eye region (F,L,R). The red dashed lines indicate the appropriate regions for comparison between control (B, H) and *Topgal*; *Lens-cre; Catnb*^*lox*(*ex3*) ^embryos. dov-dorsal optic vesicle, ve-ventral ectoderm, wf-whisker follicles, sv-sensory vibrissae, prpe-presumptive retinal epithelium.

To address this issue further, we have combined the *Lens-cre*; *Catnb*^*lox*(*ex3*) ^alleles with the *Topgal *transgene, a β-galactosidase reporter for the canonical *Wnt *pathway [6]. In control embryos, *Topgal *expression is not observed in the presumptive lens ectoderm, periocular ectoderm or optic vesicle at E9.5 (Fig. 2A,B,C). Low levels of *Topgal *expression are observed in the presumptive retinal pigmented epithelial (RPE) layer of the dorsal optic vesicle by E10.5 (Fig. 2H) but remains absent from the lens pit and remainder of the optic cup (Fig. 2I). In E12.0 wild-type embryos, *Topgal *expression is observed in the presumptive conjunctival epithelium (Fig. 2N), scattered cells in the developing RPE and the follicles for the sensory vibrissae and whiskers (Fig. 2M,N). Sectioning also reveals a few *Topgal *positive cells within the presumptive retina (Fig. 2O). As might be expected, and in contrast with wild-type embryos, *Lens-cre*; *Catnb*^*lox*(*ex3*)^; *Topgal *embryos show X-gal labeling in the presumptive lens. At E9.5, this appears as a few scattered cells (Fig. 2E and 2F) but by E10.5, the staining is intense and occupies the entire region of *Lens-cre *expressing ocular ectoderm (Fig. 2K, L). At both E10.5 and E12.0, the ectoderm of the naso-lacrimal groove remains *Topgal *expressing (Fig. 2K and 2Q). Unlike wild-type embryos (Fig. 2O), mutant embryos at E12.0 do not show *Topgal *expressing cells within the optic cup either within the pigmented, RPE-like regions or within presumptive retina-like layers (Fig. 2R). Expression of the *Topgal *transgene in the *Lens-cre *expressing regions of *Catnb*^*lox*(*ex3*) ^embryos suggests that the canonical *Wnt *pathway is being activated. At E12.0, wild-type embryos have facial structures with high levels of Wnt signaling according to TOPGAL expression (Fig. 2M and 2N) including the whisker barrels and upper mandible. These structures are absent or reduced in the mutant (Fig. 2P and 2Q) because earlier expression of the β-catenin gain-of-function allele has prevented or perturbed their development. Using antibodies specific for the C-terminal and exon 3 regions of β-catenin, we have previously shown that the *Lens-cre*; *Catnb*^*lox*(*ex3*)^combination of alleles gives the expected upregulation of nuclear β-catenin in ocular region ectoderm [32]. Combined, these data suggest that activation of the *Wnt *pathway is responsible for profound defects in development of the eye and other facial structures in *Lens-cre*; *Catnb*^*lox*(*ex3*) ^embryos.

Consistent with observed embryonic defects, adult *Lens-cre*; *Catnb*^*lox*(*ex3*) ^mice show major defects in development of the eye and facial structures. As seen in Fig. 3A and 3B, they are unable to open their eyelids at weaning and have the appearance of anophthalmia. In the region where they eye should be, there is a hairless nodule at the nasal extremity of the eyelid suture (Fig. 3A and 3B; arrow). The identity of the nodule is unclear but histologically, it consists of epidermal layers with underlying connective tissue (data not shown). The snout of the *Lens-cre; Catnb*^*lox*(*ex3*) ^mice is shorter than that of wild-type littermates (Fig. 3A and 3B) and their teeth are badly misshapen and overgrown (Fig. 3C and 3D) possibly a secondary consequence of misalignment of the upper and lower incisors. In some embryos, the anterior region of the palate shows clefting (Fig. 3E and 3F). Given the appearance of older mutant animals, this likely represents a developmental delay in fusion of the palate and nasal processes. This defect may also help explain the misalignment of the upper and lower incisors. The smaller body size observed in *Lens-cre*; *Catnb*^*lox*(*ex3*) ^mice when compared to wild-type littermates may be due to poor feeding.

The fore-shortened snout and abnormal eye development are readily visible at E15.5 when it is also possible to see that the retinal pigmented epithelium (RPE) is reduced (Fig. 3G and 3H). Sections of paraffin-embedded E15.5 embryos stained with hematoxylin and eosin reveal that in the eye region, the lens, cornea and eyelids are absent, and that there is a highly convoluted, disorganized retinal tissue and retinal pigmented epithelium (Fig. 3I and 3J). Since the ocular glands, the Harderian and lacrimal glands, are derived from periocular surface ectoderm, we stained skinless P2 whole mount preparations of mouse heads with Nile Blue. This showed that the lacrimal gland was absent from its normal position adjacent to the ear (Fig. 3K and 3L). The Harderian mesenchyme is present but does not contain any branched epithelium (data not shown).

### β-*catenin *gain-of-function disrupts molecular events in early lens development

The expression of *Lens-cre *and, consequently, activation of the *Wnt *pathway via the *Catnb*^*lox*(*ex3*) ^allele, is restricted to ocular region surface ectoderm [1,32]. This is confirmed by the observation that the *Z/AP *allele is only activated by *Lens-cre *in this region (Fig. 1). Combined, these observations imply that patterning defects in the optic cup are likely an indirect result of cell fate changes in the surface ectoderm.

**Figure 4:**
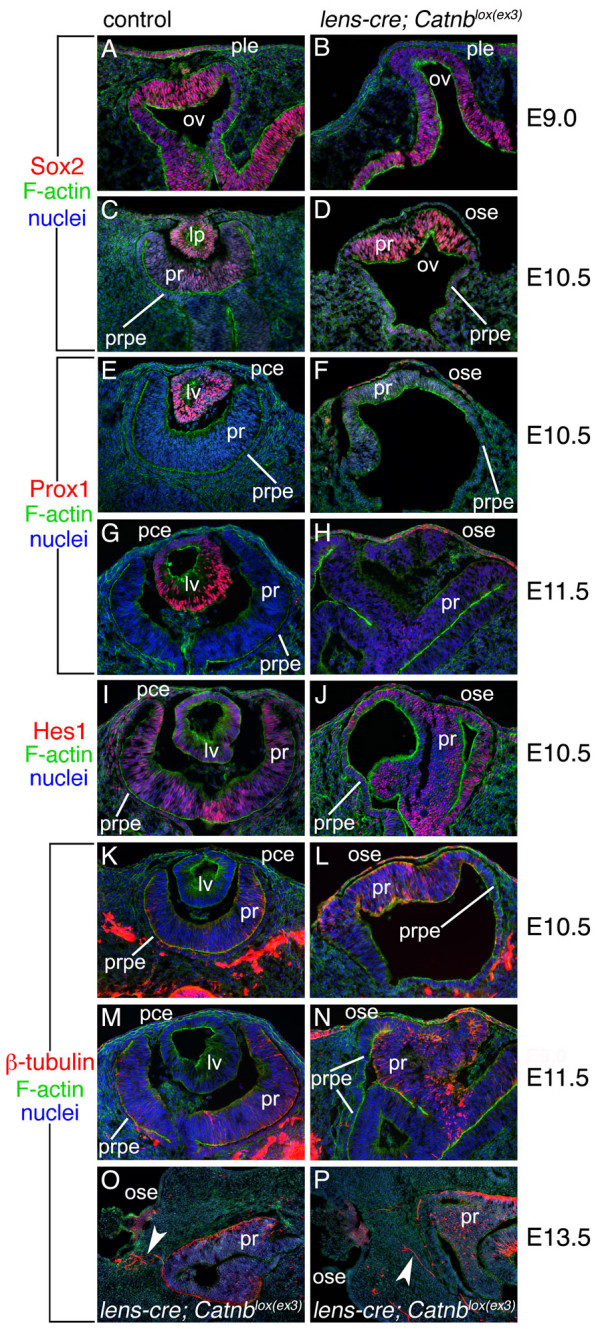
**Lens fate is lost in the central ocular ectoderm of β-catenin gain-of-function mice**. Cryosections of the indicated embryonic age and genotype showing Sox2 (A-D), Prox1 (E-H), Hes1 (I, J) and β-tubulin (K-P) immunolabeling (red). Labeling for F-actin is also shown (green) with Hoechst 33258 nuclear labeling (blue). Arrowhead indicates processes extending from presumptive retina of *Lens-cre*; *Catnb*^*lox*(*ex3*) ^mutant mice that are positive for β-tubulin immunolabeling. Labeling abbreviations as in Fig. 1.

We have previously shown that in *Lens-cre*; *Catnb*^*lox*(*ex3*) ^mutant embryos [32], the lens does not develop and that the lens markers Pax6 [9,13] and AP2α [38] are down-regulated. To understand the molecular phenotype in more detail, we have examined the ocular region for the expression of additional lens proteins, Sox2 and Prox1. Sox2 is an HMG family, *Sry*-related transcription factor that has been implicated in lens development because it can associate with Pax6 and in this complex can regulate the expression of crystallin genes [19,20]. Sox2 is normally expressed in both presumptive lens and retina from an early stage of eye development and therefore may have additional activities besides regulating crystallin expression. In wild-type embryos at E9.0, Sox2 is highly expressed in the presumptive lens ectoderm, presumptive retina, optic stalk, neural tube and at lower levels in the presumptive RPE (Fig. 4A). By E10.5 as the lens pit and optic cup have undergone a coordinated invagination, Sox2 expression remains high in the lens pit but is reduced in presumptive retina (Fig. 4C). By contrast, activation of the *Wnt *pathway with the *Lens-cre*; *Catnb*^*lox*(*ex3*) ^combination results in the reduction or absence Sox2 immunoreactivity in both presumptive lens and retina at E9.0 (Fig. 4B). By E10.5, Sox2 remains absent from the presumptive lens ectoderm but is upregulated in presumptive retina (Fig. 4D). The absence of Sox2 from the presumptive lens is consistent with the absence of lens formation.

As would be anticipated [39], immunostaining for Prox1 is strong in the lens vesicle of E10.5 and E11.5 control embryos but is not present in the surface ectoderm overlying the lens (Fig. 4E and 4G) in mutant embryos. Although the lens does not form in the *Lens-cre*; *Catnb*^*lox*(*ex3*) ^mutant embryos, Prox1 immunolabeling is detected cells of the surface ectoderm overlying the malformed retinal structure at E10.5 (Fig. 4F), E11.5 (Fig. 4H), and E12.5 (data not shown). Prox1 is an important lens fate marker and is necessary for the differentiation of lens fiber cells [39]. Prox1 is also a marker for neural cells and is required for retinal cell fate and in particular, the formation of amacrine and horizontal cells [8].

During retinal development, precursors give rise to several different types of cells at various stages of embryogenesis and postnatal development [4,29]. Previous studies have shown that Hes1 plays an important role in maintenance of retinal precursors and postnatal differentiation of retinal ganglion cells [2,10,34]. Hes1 is also expressed in the lens [23]. In control embryos at E10.5 (Fig. 4I), Hes1 immunostaining is detected in the lens vesicle and in presumptive retina. In *Lens-cre*; *Catnb*^*lox*(*ex3*) ^embryos, the distribution of Hes1 in the layers of the abnormally shaped retina is similar, but as with Prox1, only a few cells of the ocular surface ectoderm exhibit expression (Fig. 4J).

In β*-catenin *gain-of-function surface ectoderm, cell death was found to be normal using TUNEL labeling (data not shown) which, coupled with the sporadic distribution of Prox1 and Hes1 in this region, tends to suggest that these ectodermal cells may have adopted multiple cell fates. The absence of Pax6 [32] and Sox2 and the complete failure of lens formation suggest that lens fate is not one of these. Since Prox1 and Hes1 are markers for both lens and neural tissues, their expression hinted at the possibility of a neural fate change. To test this directly, we labeled control and mutant eye tissues for the neural-specific marker β-tubulin. The surface ectoderm from *Lens-cre*; *Catnb*^*lox*(*ex3*) ^mutant embryos is positive for β-tubulin beginning at E10.5 (Fig. 4L) while neither the surface ectoderm nor the lens vesicle of the control are labeled (Fig. 4K). As might be expected, the retina stains positively for β-tubulin in both control and mutant embryos at E10.5 and E11.5 (Fig. 4K–N). We also have observed mutant embryos that expression of β-tubulin begins earlier at E9.5 (data not shown). By E13.5, β-tubulin is detected in processes (white arrowhead) that appear to extend from the malformed retinal structure to the overlying epithelium that also expresses β-tubulin (Fig. 4O and 4P). These may represent the misguided axons of ganglion cells that develop in the abnormal retina.

### Optic cup patterning is defective in β-*catenin *gain-of-function embryos

We noted that in the β*-catenin *gain-of-function mutants, though the conditional up-regulation of *Wnt *signaling was restricted to ocular region surface ectoderm, the underlying optic vesicle developed abnormally. Using immunohistochemistry to investigate this aspect of the mutant embryos further, we characterized formation of the optic vesicle using two well-characterized markers, Chx10 and Mitf, which identify presumptive neural retina and RPE, respectively. Chx10 is the earliest known marker specifically expressed in the neuroretina [24] and is upregulated in the central distal optic vesicle at E9.5. Mitf is crucial for the development of RPE identity [16] and is expressed throughout the optic vesicle at E9.0 [30]. At E9.5 Mitf is down-regulated in the presumptive neuroretina [30] when Chx10 expression is first observed. It has been suggested that Chx10 represses *Mitf *expression to regulate patterning of the optic cup into the retinal and pigmented epithelial layers [17].

**Figure 5:**
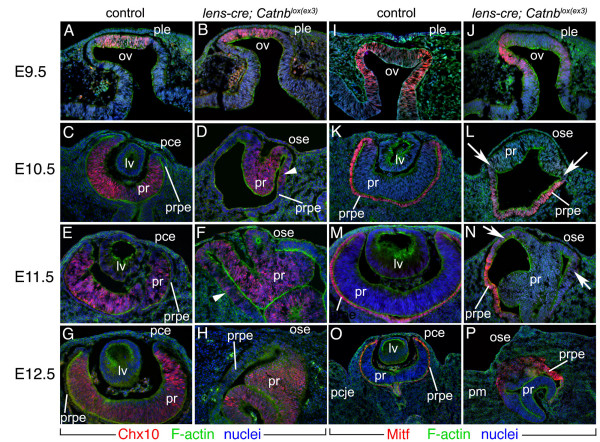
**Expression of retinal epithelium and RPE markers in β-catenin gain-of-function embryos**. Immunolabeling for Chx10 (red; A-H) and Mitf (red; I-P) on cryosections from control (A, C, E, G, I, K, M, O) and *Lens-cre*; *Catnb*^*lox*(*ex3*) ^mutant (B, D, F, H, J, L, N, P) embryos at the ages indicated. Immunofluorescence for F-actin is also shown (green) with Hoechst 33258 nuclear labeling (blue). White arrowheads in (D, F) indicate area of expanded Chx10 expression. (L, N) White arrows indicate areas with diminished Mitf activity. Labeling abbreviations as in Fig. 1.

At E9.5, the pattern of Chx10 immunolabeling in control and mutant embryos is identical (Fig. 5A and 5B) with signal apparent in the distal, central optic vesicle. By E10.5, control embryos show expression of Chx10 in presumptive retina (Fig. 5C). Expression is largely absent from the distal retinal rim and completely absent from the forming RPE. In *Lens-cre*; *Catnb*^*lox*(*ex3*) ^mutants, despite the absence of lens structures, central presumptive retina expresses Chx10 in a pattern that appears largely normal (Fig. 5D). However, Chx10 expression is also found in epithelium that is presumptive RPE based on its position as the proximal layer of the optic cup (Fig. 5D, white arrow). In control embryos, by E10.5, the presumptive RPE has decreased in width (Fig. 5C). Mutant embryos do not show this change and presumptive RPE appears to remain at the width it had already acquired by E9.5 (compare Figs. 5B with 5D). Abnormal expression of Chx10 and an unusually thick proximal optic cup layer is also apparent in mutant embryos at E11.5 (Fig. 5E and 5F). By this stage, the optic cup is highly convoluted. One day later (Fig. 5H) the optic cup epithelium has lost contact with the surface ectoderm, perhaps because periocular mesenchyme has migrated between the two structures.

The morphological and Chx10 gene expression changes in the optic cup suggested that RPE-retina boundary decisions are disrupted in the β-catenin gain-of-function mutant. We examined this issue further with labeling for the RPE marker Mitf [30]. At E9.5, the expression pattern of Mitf in mutant embryos appears identical to that of the control (Fig. 5I and 5J). At E10.5, Mitf is expressed throughout the developing RPE but is also observed in the very anterior retinal rim in the region from which the iris diaphragm and ciliary process will develop (Fig. 5K). The pattern of Mitf expression in the mutant optic cup is quite distinct in that it is absent from the anterior retinal rim and indeed diminishes in level well before the RPE-retinal junction (Fig. 5L; white arrow). This trend continues at E11.5 when epithelium that is arguably RPE based on Mitf expression that is negative or reduced (Fig. 5N; white arrow). By E12.5, the optic cup has lost its orientation and the Mitf positive epithelium is facing the surface ectoderm, but separated from it by periocular mesenchyme (Fig. 5P). These observed changes are consistent with the idea that activation of the canonical *Wnt *pathway in the ocular surface ectoderm has consequences for development of the other components of the eye.

In this report we describe a series of developmental defects that arise in mice that employ the *Lens-cre *transgene [1] to activate the gain-of-function β*-catenin *allele [15]. The tissue specificity of *Lens-cre *is provided by the ectoderm enhancer from *Pax6 *[7,21,40,41]. This is a small region of the 5' flanking region of the gene that in *lacz *reporter transgenes was shown to have activity in the ocular region ectoderm that would give rise to the entire lens as well as the epithelia of the cornea, conjunctiva, lacrimal gland and Harderian gland duct [12,21,28,40,41]. It was therefore unexpected that in *Lens-cre*; *Catnb*^*lox*(*ex3*) ^mutant embryos, there were extensive defects in the embryonic head in a region that encompassed nasal as well as ocular structures. Fate-mapping with *Lens-cre *and the *Z/AP *reporter indicated that a number of cell populations in the nasal region including the whisker barrels (for example, at E14.5) are derived from cells that at some point in development expressed *Lens-cre*. When combined with the observation that these cells do not apparently express ectoderm enhancer-derived transgenes [1,7,21,40,41], we can suggest that these populations may have migrated from adjacent Pax6-expressing regions and in doing so, down-regulated Pax6. Further analysis will be required to assess this proposal.

The degree to which development of the optic cup was affected by changes in the adjacent presumptive lens ectoderm was unanticipated. In a number of mutant mice where *Lens-cre *has been used for conditional gene deletions, the consequences for eye development are relatively mild. For example, conditional deletion of *Pax6 *using *Lens-cre *results in a complete failure of lens development, but other than infolding of the retina, patterning of the optic cup into retinal and RPE layers appears normal [1]. By contrast, activation of the canonical *Wnt *pathway in the presumptive lens ectoderm has severe consequences culminating in misorientation of the optic cup so that the Mitf-positive-RPE-like epithelium is facing the surface ectoderm. This dramatic change appears to be coupled with failure of normal optic cup patterning into retinal and RPE regions. In particular, the normal boundary between retinal Chx10 and RPE Mitf expression is disrupted; Chx10 is found in epithelium that would normally be RPE and Mitf is not detected in the anterior rim of the optic cup as usual. Altogether, this suggests that changes in the surface ectoderm have influenced patterning events in the optic cup and emphasizes the importance of the interaction between presumptive lens and retina.

To date, our understanding of the signaling required for the inductive interaction between presumptive lens and retina is somewhat limited, but genetic and embryological manipulations have implicated Fgf pathways [9,30]*Bmp7 *[37] and *Bmp4 *[11]. In particular, there is evidence that patterning from the optic cup is influenced by Fgf signals that have their origin in the surface ectoderm [18,30]. Specifically, it has been shown that Chx10 expression is lost if the presumptive lens is removed, but is rescued if Fgf ligands are provided [30]. Since Chx10 regulates expression of *Mitf *[17], Fgf signals are believed to indirectly pattern the optic cup. This may imply that in *Lens-cre*; *Catnb*^*lox*(*ex3*) ^embryos, this optic cup signaling is lost. It is also possible that the neural fate change observed in some cells of the presumptive lens region could result in the production of abnormal signaling factors that disrupt patterning of the optic cup. We also cannot exclude the possibility that defects in patterning of the optic cup are partly a result of abnormal responses in periocular mesenchyme as influenced by cell fate changes in the surface ectoderm.

## Conclusion

Activation of the canonical *Wnt *pathway and the presumptive lens ectoderm resulted in failure of lens formation, aberrant patterning of the optic cup and malformation of craniofacial structures. Since the surface ectoderm in the *Lens-cre*; *Catnb*^*lox*(*ex3*) ^animals never acquires the ability to form lens, the neural-like fate that is detected in these cells may disrupt the normal exchange of signals and consequently, formation of a non-functional eye. In conclusion, interactions between the surface ectoderm and subsequently the lens, with the optic vesicle and periocular mesenchyme are likely to play an important role in optic cup patterning.

## Methods

### Generation, maintenance and genotyping of transgenic mice

The following transgenic mice were used in this study: *Topgal *[6]; β*-catenin *gain-of-function (*Catnb*^*lox*(*ex3*)^; [15]); *Lens-Cre *[1]; *Z/AP *[25]. The β*-catenin *gain-of-function mice were crossed to the *Lens-Cre *mice to generate conditional mice where β-catenin would be activated in the lens placode upon the lens-specific expression of Cre-recombinase. Animals were housed in a pathogen-free vivarium in accordance with institutional policies. Gestational age was determined through detection of a vaginal plug and head/rump length measurements. At specific gestational ages, fetuses were removed by hysterectomy after the dams had been anesthetized with isofluorane.

For genotyping, yolk sacs from staged embryos were digested overnight at 55°C in tail buffer (50 mM Tris, pH 8.0; 0.1 M EDTA; 0.5% SDS) containing 100 μg/ml proteinase K. Genomic DNA was extracted using phenol:chloroform. Primers for the genotyping of β*-catenin *gain-of-function (GOF) mice were designed as reported in [15] while the PCR cycle is as follows: 95°C-4', {98°C-20", 65°C-1'} × 30, 72°C-7', 4°C. Primers for detection of the Cre-recombinase gene were generated according to [3] and used with the following PCR cycle: 95°C-4', {94°C-30", 56°C-30", 72°C-45'} × 27, 72°C-7', 4°C.

### Histology and Immunohistochemistry

#### Nile Blue Staining

Embryos at postnatal day 2 were perfusion-fixed with 4% paraformaldehyde and then placed in 4% PFA at 4°C overnight. Next, the heads of the embryos were removed and incubated in Nile blue at room temperature on a rotator. After 20 minutes, the embryos were washed with twice with 1 × PBS for 10 minutes.

#### X-gal Staining

Whole-mount embryos expressing *LacZ *reporter genes were fixed for 30 minutes using X-gal fixative (1% Formaldehyde, 0.2% Glutaraldehyde, 2 mM MgCl_2_, 5 mM EGTA, and 0.01% NP-40) and washed twice with 1 × PBS/0.02% NP-40 for 15 minutes. Embryos were then stained with X-gal solution (5 mM K_3_Fe(CN)_6_, 5 mM K_4_Fe(CN)_6_, 1 M MgCl_2_, 0.01% NP-40, 1 mg/ml X-gal) overnight at 37°C, post-fixed with 4% paraformaldehyde for 1 hour, cryoprotected in 30% sucrose-PBS, and 10 μM sections prepared.

#### Alkaline Phosphatase (AP) Staining

Whole-mount embryos expressing the *Z/AP *(*lacZ*/human placental alkaline phosphatase) reporter gene were fixed for 30 minutes on ice using the X-gal fixative described above and washed 3 times for 5 minutes each with PBS/0.02% NP-40. Embryos were incubated in wash solution for 30 minutes at 75°C to inactivate endogenous alkaline phosphatase. After washing for 5 minutes in PBS/0.02% NP-40, embryos were washed in alkaline buffer #1 (100 mM Tris-HCl, pH 9.5; 100 mM NaCl; 100 mM MgCl_2_) for 10 minutes and then stained with BM purple AP substrate for 0.5–36 hours at 4°C. Using AP buffer #2 (PBS/0.1% Tween 20; 2 mM MgCl_2_), embryos were washed 3 times for 10 minutes each. For some experiments, 10 μm cryosections were then prepared.

### Immunofluorescence

For cryosectioning, embryos were fixed with 4% paraformaldehyde, cryoprotected in 15% and then 30% sucrose, and 10 μM frozen sections prepared. Sections were rehydrated in PBS with 0.1% Tween before incubation in blocking solution (4% milk in TST {10 mM Tris-HCl, pH 7.4; 150 mM NaCl; 0.1% Tween20}) for 45 minutes. Sections prepared for Chx10 antibody staining were permeabilized with 1 × PBS/1% Triton X-100 for 20 minutes prior to blocking. Overnight primary antibody incubation was done at room temperature followed by secondary antibody incubation for 30 minutes. Primary antibodies with dilutions used are as follows: polyclonal rabbit anti-Chx10 (1:750; gift from R. Chow), polyclonal rabbit anti-Prox-1 (1:5000; Chemicon), polyclonal rabbit anti-Sox2 (1:1000; Chemicon), polyclonal Mitf-1 (1:2500; gift from H. Arnheiter), polyclonal rabbit β-tubulin (1:500; Abcam), polyclonal rabbit Hes1 (1:1000; NL. Brown). Alexa Fluor secondary antibodies and Alexa phalloidins were obtained from Molecular Probes and used at a 1:1000 dilution (#A-11072, #A11020, #A-11070, #A-11017, #A-12381). All sections were counterstained with Hoechst 33342 (Sigma, #B-2261) for visualization of nuclei.

### Preparation of paraffin sections

For paraffin sectioning, embryos were fixed in 4% paraformaldehyde, dehydrated in ascending series of ethanols, infiltrated and embedded with paraffin. Using a Leica RM2145 microtome, 4 μm sections were prepared. Paraffin sections were prepared and stained for hematoxylin and eosin using a conventional method [5].
